# The validation of the Arabic version of the resilience scale 14 (RS-14)

**DOI:** 10.1186/s12912-023-01392-9

**Published:** 2023-07-11

**Authors:** Gladys Honein-AbouHaidar, Imad Bou-Hamad, Suzanne Dhaini, Patricia Davidson, Nancy R Reynolds, Ibtisam M Al-Zaru, Muntaha Gharaibeh, Nuhad Yazbik Dumit

**Affiliations:** 1 Hariri School of Nursing, American University of Beirut, Bliss Street, Riad El Solh 1107, PO Box: 11 0236, Beirut, 2020 Lebanon; 2 Department of Business Information and Decision Systems, Suliman S. Olayan School of Business, American University of Beirut, Bliss Street, Riad El Solh, PO Box: 11 0236, Beirut, 1107 2020 Lebanon; 3 Institute of Nursing Science, Department Public Health, Faculty of Medicine, University of Basel, Bernoullistrasse 28, Basel, CH-4056 Switzerland; 4 University of Wollongong, Wollongong, Wollongong, New South Wales, NSW 2522 Australia; 5 Johns Hopkins School of Nursing, 525 N Wolfe St, Baltimore, MD 21205 USA; 6 Jordan University of Science and Technology, Ar Ramtha 3030 Ar-Ramtha, Irbid, Jordan

**Keywords:** Arabic, Nurses, Resilience, Scale, Validity.

## Abstract

**Background/Objectives:**

Nurses in Lebanon are facing multiple crises and the severity of the situation calls for an empirical examination of their resilience status. Evidence indicates that resilience can buffer the negative effect of workplace stressors on nurses and is associated with favorable patient outcomes. The objective of this study was to test the psychometric properties of the Arabic Resilience Scale-14 that was utilized to measure resilience among Lebanese nurses,

**Methods:**

Data was collected from nurses working in health care centers using a cross-sectional survey design. We estimated the confirmatory factor analysis using the Diagonally Weighted least Squares. Fit indices for the confirmatory factor analysis model included Model chi-square, root-mean squared error of approximation and Standardized Root Mean Square Residual. Statistical significance was set at p < 0.05.

**Results:**

1,488 nurses were included in the analysis. The squared multiple correlations values ranged from 0.60 to 0.97 thus supporting the construct validity of the originally hypothesized five factor model (self-reliance, purpose, equanimity, perseverance, and authenticity).

**Conclusions:**

The Arabic version of the Resilience Scale 14 tool is considered a valid tool for measuring resilience in any situation involving Arabic speaking nurses.

## Background

Stress due to adverse work environment is a major concern for the nursing profession. Common adverse work environment stressors include increased workload, shift work, poor interpersonal relationships, pressure to deliver quality care, competing demands, and death and dying [1]. These stressors jeopardize nurses’ physical and mental well-being, quality of caregiving to patients, and professional retention. Nurses often report fatigue, lack of sleep, increase in smoking, inability to maintain a physically active lifestyle [2]. Mental exhaustion is expressed as fatigue, irritability, lack of concentration, unhappiness, depressive symptoms, and depersonalization [3]. Their passion for their profession gradually erodes leading to compassion fatigue [4] and inability to work effectively [5], and to an increased risk to patient safety [6, 7]. The high level of burnout [8] and the emotional exhaustion accruing overtime [9] lead some nurses to dropout from the nursing profession. Despite reported work stress and nurses’ inabilities to work effectively and its bearing on patient safety, many nurses remain resilient; the ability to maintain personal and professional well-being despite work stress and substantial adversities [3].

Resilience refers to the “adaptation to stress, balance, competence, determination, optimism and acceptance” [10]. There are individual characteristics that define resilient nurses, including ability to cope and manage stress resulting from physical, mental and emotional nature of their work; self-efficacy in overcoming challenges; positive outlook on life matters; and existence of sense of humor and a high morale [11]. These characteristics help nurses face adversities at work and adopt successful problem skills to overcome their challenges [3]. Evidence shows that resilience acts as a mediator between emotional exhaustion and stress and burnout [12, 13]. The higher the level of resilience among nurses, the less the stress, burnout, and the better the quality of care and retention rates [13–15].

In Lebanon, nurses are at a high risk for resilience depletion for various reasons. There is an entrenched shortage in nursing workforce despite all the efforts to increase recruitment and retention [16]. Compared to international nurse density, Lebanon remains among the lowest: with 1.7 nurse density per 1000 population compared to 4.49 per 1000 population in Europe and North American in 2018 [17]. The situation was exacerbated with the influx of nearly 2 million Syrian Refugees since 2011 [18], many of them presenting with serious health care needs [14, 19–21], this translated into an exponential increase in workload on nurses. As a result, nurses voiced an increase in burnout symptoms, loss of resilience and further reduction in job satisfaction; and importantly, threatened the quality of patient care [22]. Further, although this study was conducted just before the multiple major events hit the country and led its economy to be on the brink of collapse, the nursing workforce shortage got even worse with many nurses laid off from work. Yet, many nurses remain resilient while others are ready to leave the profession. The severity of the situation calls for an empirical examination of the resilience status of nurses in Lebanon in order to intervene with appropriate measures to improve the nurses’ wellbeing, their quality of care and their retention in Lebanon [23, 24]. Hence, the purpose of the current study was to utilize an Arabic translation of the Resilience Scale 14 (RS-14) on a representative sample of nurses working in hospitals and primary health care centers in Lebanon to objectively measure resilience, while concurrently testing the psychometric properties of the Arabic RS-14.

## Methods

This study is part of a larger project, the PROfILE study, (Nurses’ perspectives on the Syrian refugee healthcare in Lebanon and Jordan) [25]. The parent study aims to: (a) explore nurses’ perspectives on the effect of the influx in healthcare demands due to the protracted Syrian refugee crisis in Lebanon and Jordan, and, (b) to examine how this increase in demand is affecting the quality of care services using several previously validated scales. In this study, we use the results from Lebanon only with the aim of validating the Arabic version of the Resilience Scale 14 (RS-14).

We used a cross-sectional survey data collected as part of PROFILE parent study. The settings where the participants were invited to partake in this study were registered nurses working in hospitals and primary healthcare centers. For full details on the PROFILE study protocol, please refer to the publication [25].

### Participants

All nurses licensed by the relevant national health authority were considered eligible to participate in the study. We only included those who provided direct health care services. The following inclusion and exclusion criteria were adopted [25]:

Inclusion:

- Nurses working in hospitals and primary healthcare centers.
- Nurses providing direct patient care in hosting communities for at least one full month and work for at least 8 h/ week.

Exclusion:

- Nurses in academia and other industries.
- Nursing students, nursing instructors, and volunteers.
- Nurses who assume managerial/administrative positions and are involved in indirect service.
- Nurses who are not involved in providing service to Syrian refugees.

There were approximately 3,000 nurses that provided health care services, of which 1566 nurses filled the questionnaire.

As our aim was to validate an instrument using a confirmatory factor analysis (CFA), the minimum sample size needed was 140 participants. For 14 item scales, the minimum recommended item-ratio for CFA is 10 subjects for each parameter [26, 27]. Given that the parent study used multiple scales, our sample size exceeded the minimum required for validating the RS-14 scale.

### Survey instrument

The survey instrument included items measuring nurses’ socio demographic characteristics, organizational and work-related factors, and the 14-item scale.

#### Nurses’ socio-demographic characteristics

Socio-demographic characteristics included age (by decade, 20 - > 50 years), gender (dichotomous), marital status (categorical), highest educational attainment (categorical, Technical Nursing Diploma, Bachelor in Nursing, Masters in Nursing), and monthly income in United States dollars (USD) (< 1000; >1000 USD).

#### Organizational variables

Organization was measured with three items: geographic area, type of service and location of service. *Geographical area* was measured as served or underserved as described by El Jardali and colleagues [28]. Underserved areas are defined as those having limited resources and access to healthcare services, educational attainment, safe infrastructure (water, electricity, roads, etc.) and economic sources in addition to poor population indicators such as remote, urban slums, refugee camps, conflict zones, and severely affected by man-made or natural disasters [28]. *Type of service* was measured as hospital or Primary Health Care setting. The *location of the service* was governorate of Lebanon (Akkar, Baalbeck-Hermel, Beruit, Bequaa, Keserwan-Jbeil, Mount Lebanon, Nabatieh, North, or South).

#### Work-related variables

Work-related variables included *number of hours* worked per week (categorical ± or equal to 42.5); *type of shift work for hospital nurses* (day, evening, night); and *whether they were providing nursing care to refugees* at their units/centers (yes, no); and *for how long* (1 to 2 years, 3 to 4 years, or equal to or greater than 5 years).

#### Resilience scale (RS-14)

Several resilience scales, at least 15, are often used in the literature [29]. Those that demonstrated the best psychometric ratings were three: the Connor-Davidson Resilience Scale, the Resilience Scale, and the Resilience Scale for Adults. A recent study [30] used and validated the Connor-Davidson Resilience scale among a group of women in Lebanon. The Resilience Scale for Adults used to assess the ability to bounce back or recover from stress [31], which does not apply to our situation as nurses were still amid the crises. Thus, we were left with the Resilience Scale.

The Resilience Scale 25 (RS-25) is a widely used measure because of its appropriateness for use with respondents of different ages and populations including medical staff, and for its proven internal consistency [32]. Developed after narratives of women who expressed resilience, the original Resilience Scale (RS) was originally comprised of 50 items. It was later reduced to 25 item and later to 14 items [33, 34]. The RS-14 item scale has been validated in numerous languages: Chinese, English, Portuguese, Finnish, Korean, Spanish, Urdu, Italian, Greek, and Taiwanese [33, 35–39], Polish [40, 41], French [42], and Lithuanian [43], Swedish and Japanese languages [44, 45] including Arabic [46]. In 2016, Wagnild updated the scale [34].

The RS-14 scale was used in this study with permission from the author, Gail M. Wagnild, through a licensed agreement. The scale in both English and Arabic languages cannot be transferred as per this agreement. The updated RS-14 item scale version used in the current study and being validated in Arabic language [32] includes 11 items from the older RS-14 scale and three modified items. (J.3- “I usually take things in stride” changed into “I usually take things calmly and evenly when bad unexpected things happen; J.11- changed from: “I can get through difficult times because I’ve experienced difficulty before” into “I can usually look at a situation in a number of ways”; and J14 from “In an emergency, I am someone people generally rely on” into “I have enough energy to do what I have to do”. These three items were changed based on piloting the questionnaire with 10 nurses who were excluded from the sample. At least five nurses of the 10 suggested the modifications. Accordingly, the research team deliberated the suggestions and made modifications. Item J3 was rephrased, yet kept the same meaning. Items J11 and J 14 were taken from the longer version of the scale, RS™ that has 25 items. The RS™ was translated into Arabic and used in a study in Lebanon [47], however was not psychometrically tested.

The 14 items contain five constructs: (1) *self-reliance* (items 1, 5, and 13); (2) *purpose/meaningfulness* (items 2, 8, and 12); (3) *equanimity* (items 3, 9, and 11); (4) *perseverance* (items 6, 7, and 14); (5) and *authenticity* (items 4 and 10) [33, 34]. Each of these constructs refer to individuals’ reactions when facing adversity: self-reliance refers to self-efficacy in problem-solving, thus the ability accrued over lifetime experiences in comprehending and accepting own strengths and limitations; purpose/meaningfulness is believing that life has an ultimate purpose beyond the current situation, thus there is a reason for which to live; equanimity refers to a balanced and moderate response in extreme situations, this construct is often related to the sense of humor; perseverance is the capability of pursuing despite adversities; while authenticity^1^ points to each individual’s specificity reacting to the situation [10]. Items are measured on a seven-point Likert scale ranging from 1 (strongly disagree) to 7 (strongly agree).

### Procedure

#### Ethic approval

The approval of the Ethical Review Committee at the American University of Beirut was secured before the initiation of this study (IRB number is NUR.ND.15/SBS-2018-0229). The data collection occurred between October and December 2019.

#### Translation and back translation of the RS scale

In this study, the updated RS-14 scale was translated into Arabic and back translated into English independently by two health care professionals who spoke English and Arabic. The final version of the translated items was pilot tested with a sample of 10 eligible nurses who met the inclusion criteria to validate the content. They were excluded from participating in the study. The original, translated and back translated questions were then matched to ensure accuracy of translation. The pilot sample was asked to elaborate on their understanding of each question; questions and items were clearly understood by participants, and thus no changes were made.

### Data analysis

First, we conducted a descriptive analysis using frequencies for categorical variables, Cronbach alpha, and convergent validity. Second, we computed (CFA), then did a listwise deletion for the missing data. Given that our data was ordinal, we used the Diagonally Weighted least Squares (DWLS), which is a method specifically designed to estimate a CFA model for ordinal data.

To confirm the construct validity of the RS-14 scale, we hypothesized a 5-factor model representing the five latent variables (self-resilience, purpose, equanimity, perseverance, authenticity) of the original scale. Fit indices for the CFA model included Model chi-square, which assesses the overall fit and the discrepancy between the sample and fitted covariance matrices. In general, a low chi-square value relative to the degrees of freedom (and a higher p-value > 5%) indicates better model fit. However, there are several drawbacks to using the chi-square statistic as a model fit index, the most notable of which is its sensitivity to sample size, with larger sample sizes resulting in a lower p-value [48]. Thus, further tests were used for validation including root-mean squared error of approximation (RMSEA, values < 0.08 are desirable), comparative fit index (CFI, values > 0.95 are desirable), and Standardized Root Mean Square Residual (SRMR, values < 0.08 are desirable) [49, 50].

Finally, we examined the variance of the observed variables in relationship to the latent variable using the squared multiple correlations (SMC) [51].

We used SPSS (version 25) and lavaan package in R software (version 4) to estimate the CFA models. Statistical significance was set at $p<0.05$.

## Results

### Nurses’ socio-demographic characteristics

The nurse participants (N = 1,566), a response rate of 52.2%. As seen in Table 1, the majority of nurses (83%) were between the ages of 20 and 40, with nearly three-quarters being females (74%). A technical nursing diploma was earned by more than half of the participants (55%) and a bachelor’s degree in nursing was held by over two-thirds (33%) of the participants. Less than two thirds of the nurses were married or engaged (58%), and 66.3% had a monthly salary of less than 1000 USD.

**Table 1:** Participants’ demographics, organizational characteristics, and work-related factors (N = 1,566)

Characteristic	n	%
**Demographic**		
Age (in years)		
20–30	696	44.4
31–40	607	38.8
41–50	179	11.4
>50	51	3.3
Missing	33	2.1
Gender		
Male	279	17.8
Female	1158	73.9
Missing	129	8.2
Highest educational attainment		
Technical Nursing Diploma (BT, TS, LT)	863	55.1
Bachelor of Science in Nursing	488	31.2
Master of Science in Nursing	91	5.8
Other*	86	5.5
Missing	38	2.4
Marital status		
Single	578	36.9
Married/engaged	901	57.6
Separated/divorced	34	2.2
Missing	41	2.6
Monthly salary income in dollars		
<1,000	1039	66.3
≥1,000	442	28.2
Missing	85	5.4
**Organizational characteristics**		
Geographical area		
Underserved	1043	66.6
Served	471	30.1
Missing	52	3.3
Type of service		
Hospital	1136	72.5
Primary health care	430	27.5
Governorate		
Beirut	220	14.0
Mount Lebanon	251	16.0
Akkar	62	4.0
North	254	16.2
Bekaa	171	10.9
South	368	23.5
Baalbeck-Hermel	101	6.4
Nabatieh	87	5.6
Missing	52	3.3
**Work-related factors**		
Number of hours worked per week		
< 42.5	493	31.5
42.5	487	31.1
>42.5	535	34.2
Missing	51	3.3
Type of shift work		
Day (including PHC)	581	37.1
Evening	56	3.6
Night	83	5.3
More than one type of shift work	448	28.6
Missing	398	25.4
Providing nursing care to Syrian refugees		
Yes	1378	88.0
No	139	8.9
Missing	49	3.1
*If yes, to providing nursing care to Syrian refugees* *How long in years*		
1–2	359	22.9
3–4	396	25.3
≥ 5	601	38.4
Missing	210	13.4

### Organizational and work-related factors

As illustrated in Table 1, two thirds of the nurses worked in an underserved area (67%), the majority of respondents worked in hospital setting (73%), one third were based in Beirut (14%) or in Mount Lebanon (16%), while the remaining were distributed across the other regions in Lebanon. The number of hours worked per week were equally distributed, with over a third (34%) working more than 42.5 h per week, and third of the participants (31%) working 42.5 h or less. Almost same proportion (37%,) worked the day shift only in both PHC and hospital settings, and 29%, served more than one shift, mainly among hospital staff. The majority of our respondents, (88%), provided nursing care to Syrian refugees, among those 38% had equal to or more than 5 years of working with Syrian refugees.

Table 2 is a display of the means, SDs, and range of responses on the 14 items of the resilience tool. The means ranged from 5.55 (J3) to 6.06 (J10), and the range from 1 to 7.

**Table 2:** Means, standard deviation, minimum and maximum of the resilience tool

Resilience indicators	Mean	SD	Min	Max	Missing values
J1	5.70	1.58	1	7	57
J2	6.01	1.41	1	7	48
J3	5.55	1.48	1	7	49
J4	5.88	1.38	1	7	48
J5	5.88	1.27	1	7	46
J6	5.95	1.30	1	7	54
J7	6.02	1.28	1	7	48
J8	6.00	1.28	1	7	46
J9	5.39	1.73	1	7	47
J10	6.06	1.23	1	7	48
J11	5.76	1.41	1	7	60
J12	6.03	1.39	1	7	47
J13	5.70	1.45	1	7	45
J14	6.01	1.25	1	7	45

### Reliability and convergence validity

As expected, the reliability of the total score was positively and significantly correlated with its subscales (Cronbach’s alpha: 0.67–0.84) (r = 0.91).

We estimated the convergent validity and calculated the average variance extracted using the R package “semTools” (AVE). Convergent validity is established statistically when the AVE is greater than 0.50.

The AVEs for the five factor models are as follow: Self-resilience (0.497, ~ 0.5); Purpose (0.635); Equanimity (0.473, ~ 0.5); Perseverance (0.641); Authenticity (0.677).

### Construct and criterion validity

Given that the RS-14 scale is based on a strong theoretical foundation, and since the developer of the scale when they conducted an exploratory factor analysis, they identified a factor structure, we deemed unnecessary to conduct an exploratory factor analysis and resorted to conducting a CFA study to the translated Arabic version of the scale.

### Confirmatory factor analysis

From 1,566, we removed 118 surveys because they had missing data in at least one variable. The final sample used in the CFA analysis included 1,448 surveys.

In Table 3, we show the fit indices for this model. Chi-square for 1 factor model was 559.93 (p-value < 0.05) while the 5-factor model was 499.24 (p-value < 0.05); CFI = 0.996, RMSEA = 0.067, and SRMR- 0.04. Thus, we established a good fit between the expected and the observed variables.

**Table 3:** Fit Indices for Confirmatory Factor Models in Overall Sample

	RMSEA	CFI	χ2	SRMR
Model (1)	0.065837	0.995198	559.93	0.04
Model (5)	0.066772	0.995702	499.24	0.04

RMSEA = Root Mean Squared Error of Approximation; CI = confidence interval; CFI = Comparative Fit Index; SRMR = Standardized Root Mean Square Residual

The covariance coefficients among the five latent variables (self-reliance, purpose, equanimity, perseverance, authenticity) are shown in Table 4. In this table we report the covariance coefficients rather than the correlation matrix. This is due to the fact that the factors are correlated (oblique), the factor loadings are regression coefficients and not correlations and as such they can be larger than one in magnitude.

**Table 4:** Covariance coefficients among the five latent variables of RS-14

Latent variable	Self-reliance	Purpose	Equanimity	Perseverance	Authenticity
Self-reliance	1				
Purpose	0.971	1			
Equanimity	1.02	0.956	1		
Perseverance	1.04	1.006	0.950	1	
Authenticity	0.977	1.026	0.996	1.000	1

Unstandardized and standardized parameter estimates (λ) presented in Table 5 represents the factor loadings by the standard error of the item.

**Table 5:** Unstandardized and standardized estimates (λ) of latent constructs & observed variables

	Manifest	Unstandardized	SE	z	P value	Standardized	SMC
**Self-reliance**	J1	1.00	0.00	NA	NA	0.61	0.62
**Self-reliance**	J5	1.25	0.03	41.55	0.00	0.77	0.41
**Self-reliance**	J13	1.18	0.03	38.40	0.00	0.73	0.47
**Purpose**	J2	1.00	0.00	NA	NA	0.77	0.41
**Purpose**	J8	1.08	0.02	60.77	0.00	0.83	0.31
**Purpose**	J12	1.04	0.02	57.	0.00	0.79	0.37
**Equanimity**	J3	1.00	0.00	NA	NA	0.70	0.51
**Equanimity**	J9	0.81	0.02	35.38	0.00	0.57	0.67
**Equanimity**	J11	1.10	0.02	53.68	0.00	0.77	0.40
**Perseverance**	J6	1.00	0.00	NA	NA	0.83	0.30
**Perseverance**	J7	0.93	0.01	72.31	0.00	0.78	0.40
**Perseverance**	J14	0.95	0.01	74.30	0.00	0.79	0.37
**Authenticity**	J4	1.00	0.00	NA	NA	0.78	0.39
**Authenticity**	J10	1.11	0.02	72.21	0.00	0.86	0.25

SE: Standard error

SMC: squared multiple correlation

**Fig. 1:**
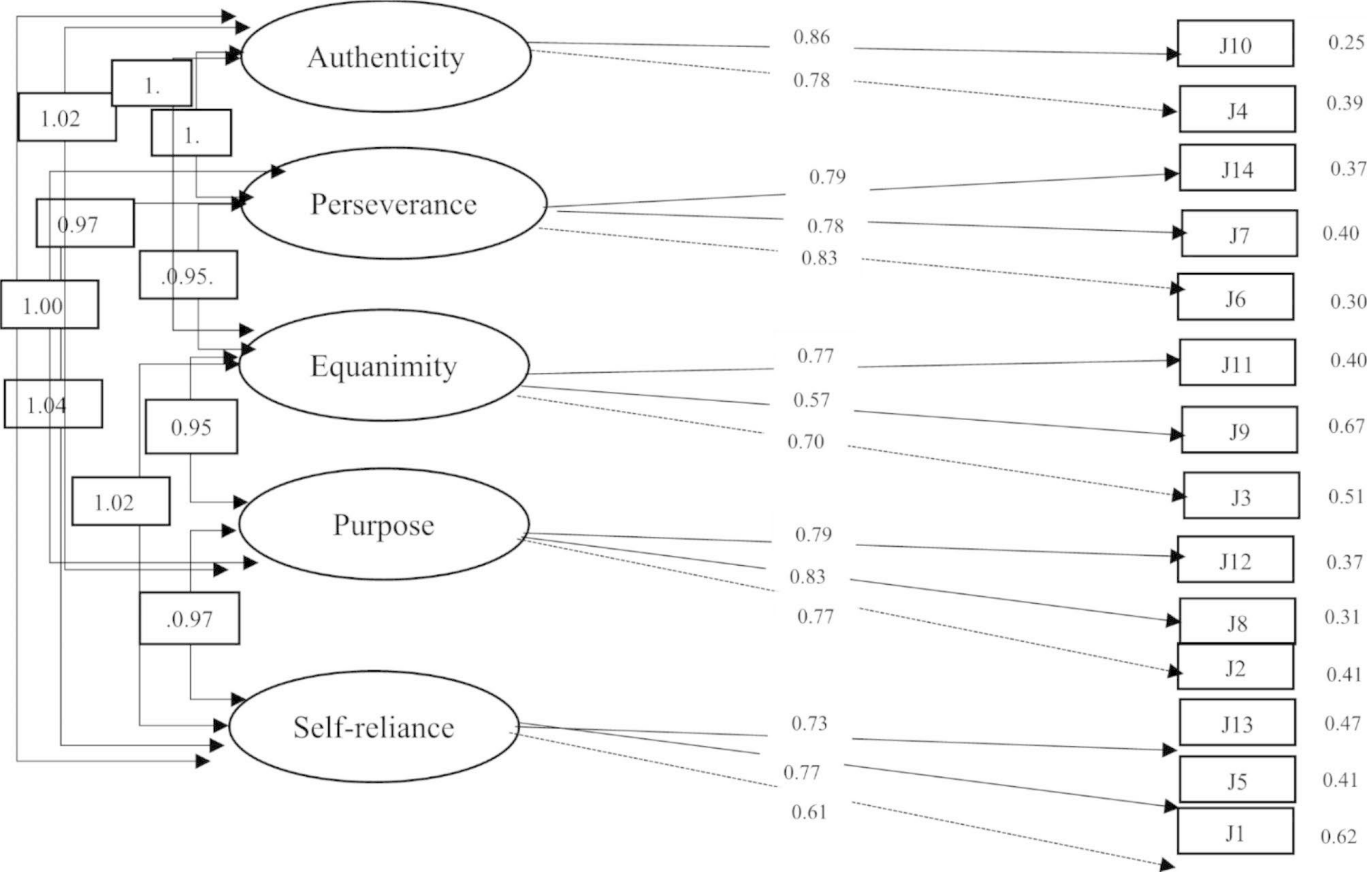
Theoretical model, the standardized parameter estimates for latent and observed variables and the SMC reliability measure

In Fig. 1, we see the theoretical model, the standardized parameter estimates for latent and observed variables and the SMC reliability measure. The squared multiple correlation (SMC) values indicating the variance of the measure ranging from 0.60 to 0.97.

## Discussion

This study used the Arabic translation of the Resilience Scale 14 (RS-14) to objectively measure resilience, while concurrently testing the psychometric properties of the Arabic RS-14 scale. The study was conducted with a representative sample of 1,488 nurses working in both PHC and hospital settings in Lebanon. Findings support the validity of the originally hypothesized five factor model (self-reliance, purpose, equanimity, perseverance, and authenticity (also labelled existential aloneness) [34]. Thus, the Arabic version can be regarded as a valid tool for measuring resilience among Arabic speaking nurses. Having five latent constructs to measure resilience has important implications. It allows the examination of each construct on resilience and provides the flexibility to address each construct individually while influencing the intra-working between them, like a gear on a bicycle, addressing one construct will eventually influence the other.

Self-reliance, defined as self-efficacy in problem-solving, is defined as the inner motivation that enables nurses to make appropriate decisions that eventually influence professional practice [52]. Self-reliance is determined by two factors: 1- when nurses have a good understanding of their job-related activities [53] and 2- have the confidence in their own ability in performing those activities (self-efficacy) [52]. Some individuals with specific characteristics, referred to in this tool as authenticity, are naturally self-motivated. Those individuals can be expected to put more effort into understanding their job-related activities and be productive independent of the work environment. If and when work shocks and stressors occur, they are likely to be less affected [52]. This perhaps explains why some nurses thrive even during crisis, what Wagnild et al. [10] refer to as perseverance, which is the capability of persevering despite adversities. Both inner motivation and perseverance accrued overtime are associated with increased resilience.

Alternatively, there are those who have little or no inner motivation (another criteria for authenticity) and are easily despaired and tend to adopt negative coping mechanisms [54]. They put less effort in understanding their job-related activities, and invest less time in their work, often translated into suboptimal performance. When compounded with work and environmental stressors, their quality of work becomes severely jeopardized and eventually engage in dysfunctional professional practices. Both lack of inner motivation and poor perseverance are associated with less resilience.

Some individuals believe that life has an ultimate purpose to live beyond the current situation or suffering, referred to in the tool as purpose/meaningfulness. When faced with adversity, those individuals draw their strength from those beliefs, which can be in the form of spiritual beliefs or belief in the importance of their roles. Other individuals’ specific reaction when exposed to adversity is to become psychologically distressed leading to helplessness and hopelessness. They may stop doing what they enjoyed doing and socializing less and unable to persevere during adversity. This withdrawal is an active decision [55]. In our sample, the mean scores for items related to purpose/meaningfulness were between 6.0 and 6.03, perhaps an indication that nurses place an important weight on their professional role during these crises, one of the reasons why they are resilient.

Equanimity refers to a balanced and moderate response in extreme situations; this construct is often related to the sense of humor. Perhaps, because Lebanese nurses have been experiencing multiple crises for over 45 years now, starting with the Lebanese civil war in 1975 until the recent Syrian crisis. These experiences may have created equanimity among the Lebanese nurses that affected their resilience.

### Implications

Efforts to boost resilience needs to be established in order to improve performance and retain nurses. These efforts can be initiated at the individual level or at the organization level. At the individual level, nurse managers need to use motivational approaches to enhance self-reliance. They need to invest more time in verbal persuasion, to meet on a regular basis with individual nurses, low and high performers alike, affirm their efforts, allow them to reflect on their weaknesses and negotiate their goals. At the organizational level, decision makers need to provide employees more support and opportunities. For example, more training opportunities to enhance their clinical and personal skills, financial and in-kind incentives, and more resources to express the gratitude of the organization for their efforts can motivate some.

Efforts to improve equanimity may include outdoor activities, physical and fun activities to create a balanced response to crisis [56]. Organizations can offer wellness programs (relaxation, mindfulness) and training on coping skills mechanisms [57]. They can provide support groups [58], strengthen the concept of meaning making of life. Spiritual activities, particularly in Arab countries where religion is an important dimension of life [46], can also improve the coping with existential suffering [56, 59].

Given the multiple crises that nurses in Lebanon are encountering, it is worth exploring whether resilience is a state or trait. Where trait refers to a stable characteristic of a person while state refers to a person’s experience in a given situation. State or trait compromises the validity and reliability of the tool used for measuring resilience. Ye et al. propose conducting test and re-test reliability and using the generalizability test to identify the items that were responsive to state interventions and thus can be used to measure the effect of resilience-based intervention studies [60].

‘Further, in order to test the responsiveness of nurses to interventions and measuring the effect of those interventions, it is also important to measure the resilience-based changes in people, it is important to conduct a minimum clinically important difference (MCID) as suggested by Ye et al. MCID is the smallest change in score that people perceive as beneficial or detrimental following a resilience-related intervention [61].

Finally, it is noteworthy to indicate that originally, the parent study has used the updated RS-14 as part of a pool of other well-established tools to examine the impact of the refugee crisis on nurses in Lebanon and Jordan. When designing the project, the focus was on the refugee crisis and its impact on the psychosocial work environment of nurses and perceived nurses and patients’ outcomes. We considered the influx of refugees to those two countries as the major strain on the health care system, specifically on nurses. We also hypothesized that resilience among nurses is the major force for keeping the health care system coping despite the challenges. But, little did we know that two major crises will hit and stretch our health care system to its brink. First, the COVID-19 pandemic, where nurses more than ever were at the frontline. Second, the August 4 Beirut blast, which ravaged the whole capital of Lebanon, killing and wounding thousands including nurses [62], and destroying infrastructure including hospitals and primary health care centers. In both crises, health care professionals, specifically nurses were at the frontline. Some paid the ultimate price, others their dedications and selflessness were beyond comparison. Thus, measuring their resilience becomes even more at the front and center of measures to examine why nurses are willing to pursue this battle despite all adversities. Though the data collection was prematurely completed before both crises, therefore we were still able to relate the outcomes to the refugee crisis in the large study.

### Strength and limitations

There are strength and limitations to this study. The main strength is we were able to survey a large number of eligible nurses in Lebanon, which is a key strength in the external validity of the results.

One of the limitations of this study is that it was based on self-reported measures and there was no mechanism for testing consistency in the responses. It is also possible that some respondents misinterpreted certain concepts while others provided socially desirable answers. Further, non-respondents were perhaps the less resilient in the workforce, as per the definition of poor resilience being less interested and motivated to act.

Another limitation of this study is the reliance on the constructs reported by Wagnild et al. The RS tool focuses on the personality characteristics that determine the capacity for resilience. The literature is replete with studies focusing on resilience and there is no agreement about the definition of resilience among scholars probably due to differences in the context and nature of the concept and the events related to it. This discrepancy resulted in different operationalization and measurements of resilience [63]. Windle, Bennett, and Noyes, reviewed 19 validated scales of resilience [29]. The authors found that the psychometric properties of these scales vary, and all have challenges regarding their psychometric properties. However, there is a consensus that resilience collectively encapsulates the phenomenon of positively adapting to adversity [64, 65]. Some conceptualized resilience as a resource, for example personal characteristics of individuals as per Wagnlid’s tool [46], others as a process [66] or as an outcome that is the individuals were able to weather the storm despite the adversities [67]. Despite the multiplicity of conceptualizations, Fisher et al. [64] affirms that there is no inherently correct or incorrect tool but rather each may “represent different aspects of the construct [64, 68]. In this study, the SR 14 Arabic version was used to understand resilience as a resource tool among Lebanese nurses. But, future studies need to use other resilience tools focusing on process and outcome in order to get a comprehensive understanding of resilience status among nurses in Lebanon.

On another note, another limitation of this study is that we adopted the classical test theory. A more precise approach could have been adopted called Item Response Theory (IRT). IRT proposes that the latent trait of participants and the properties of each item determine the probability of an individual response on a specific item. Hence, future study should be designed to provide additional information about the discriminative value of the individual items tested using an item response theory analysis [69].

Last limitation relates to challenges in translating and using tools developed in a different language than the one administered. Some of these challenges include ambiguous meaning of certain statements, using words that may not reflect the same meaning as the original one or words with multiple meanings, words bearing emotional weight [70].

## Conclusions

In summary, we demonstrated that the Arabic translation of the RS-14 tool is valid for use among the nurse’s population in Lebanon whose mother tongue is Arabic. It is worth mentioning that we needed to rephrase one item of the RS14 and replace two items with two statements from the longer version (RS25) with permission of the original author of the tool, based on participants’ feedback in the pilot phase, and as suggested by the panel of experts who are familiar with RS14 and RS25. Moreover, we suggested different approaches to improve the resilience targeting each latent variable individually and flexibly so that one construct will eventually influence the other.

## Data Availability

The data sets used and/or analyzed during the current study are available from the corresponding author on reasonable request.

## Footnotes

1 Previously, this construct was referred to as existential aloneness is the realization that each person is unique and that although some experiences can be shared, others must be faced alone.
